# Case report: horse or zebra, ascites or pseudo-ascites? Care for pictural details!

**DOI:** 10.1186/s12887-019-1826-7

**Published:** 2019-11-25

**Authors:** Alessandro Rossi, Fabiola Di Dato, Raffaele Iorio, Gianfranco Vallone, Carmine Mollica, Maria Grazia Caprio, Jean De Ville De Goyet, Maria Immacolata Spagnuolo

**Affiliations:** 1 Department of Translational Medical Science, Section of Pediatrics, University of Naples Federico II, Via Sergio Pansini n. 5, 80131 Naples, Italy; 2 Department of Advanced Biomedical Sciences, University of Naples Federico II, Naples, Italy; 3 Institute of Bio-Structures and Bio-Imaging of the National Research Council (CNR), Naples, Italy; 4 ISMETT, Mediterranean Institute for Transplants and High Specialization Therapies, Naples, Italy

**Keywords:** Pseudo-ascites, Lymphangioma, Cyst

## Abstract

**Background:**

Pseudo-ascites is a very rare condition in children and remains a challenging diagnosis. Targeted imaging may be helpful, but a high index of clinical suspicion is often necessary to guide the investigations, as pseudo-ascites may efficiently mimic true ascites. To date, still many cases of pseudo-ascites suffer diagnostic and therapeutic delay, and some are only diagnosed during surgical exploration. We report the case of a patient with a late laparoscopic diagnosis of pseudo-ascites. We retrospectively review our patient’s imaging findings and suggest new characteristic features which may help differentiate pseudo-ascites from true ascites.

**Case presentation:**

A 7-month-old infant was referred for a progressive abdominal distention. Physical examination and initial ultra-sonographic findings evoked free ascites. An extensive diagnostic workup was then performed and was negative for hepatic, renal, cardiac, intestinal, pancreatic, inflammatory or infectious diseases, malignancy and congenital metabolic disorders. Pseudo-ascites was evoked and dedicated ultra-sonographic and magnetic resonance studies were repeated but could not confirm this diagnosis. Symptomatic diuretic treatment with spironolactone and furosemide was then started. A temporary and limited effect was noted but, with time, repeated paracenteses were necessary as the abdominal distention progressed causing discomfort and breathing difficulty. Last, because the patient’s quality of life deteriorated, a peritoneal-venous shunting was proposed; as the operation started with a diagnostic laparoscopy, a benign giant cystic mesenteric lymphangioma was identified and totally excised. The resolution of symptoms was immediate and the patient remained symptom-free throughout the subsequent observation period that lasted more than 1 year.

**Conclusions:**

Increased awareness about pseudo-ascites is necessary, as the diagnosis is often overlooked, and treatment delayed. Targeted imaging may be helpful, as some specific, although not pathognomonic, features exist which may aid in the diagnosis.

## Background

Pseudo-ascites (PA) is an uncommon cause of abdominal distention mimicking ascites. It can present both in adult and pediatric patients. Its etiology comprises benign and, very rarely, malignant cystic masses that can be mis-diagnosed for ascites when they are very large. The most common entities are cystic lymphangiomas, mesenteric and omental cysts, renal cysts, cystadenomas and ovarian cysts, mesothelial cysts, enteric cysts, mature cystic teratomas, and pancreatic or traumatic cysts. Sometimes a PA can be secondary to an infection, as for example of hydatid or mycobacterial origin. Because the diagnosis of a PA is usually not evoked primarily, and because a positive diagnosis is difficult, most PA patients experience a significant delay in the identification of their condition, with a variable impairment of their quality of life until an adequate treatment (being usually a straight radical resection of the lesion) [1, 2]. We report the case of a child with a late diagnosis of PA, emphasizing the importance of the imaging findings, and suggest new characteristic imaging features which may help differentiate pseudo-ascites from true ascites.

## Case presentation

**Fig. 1:**
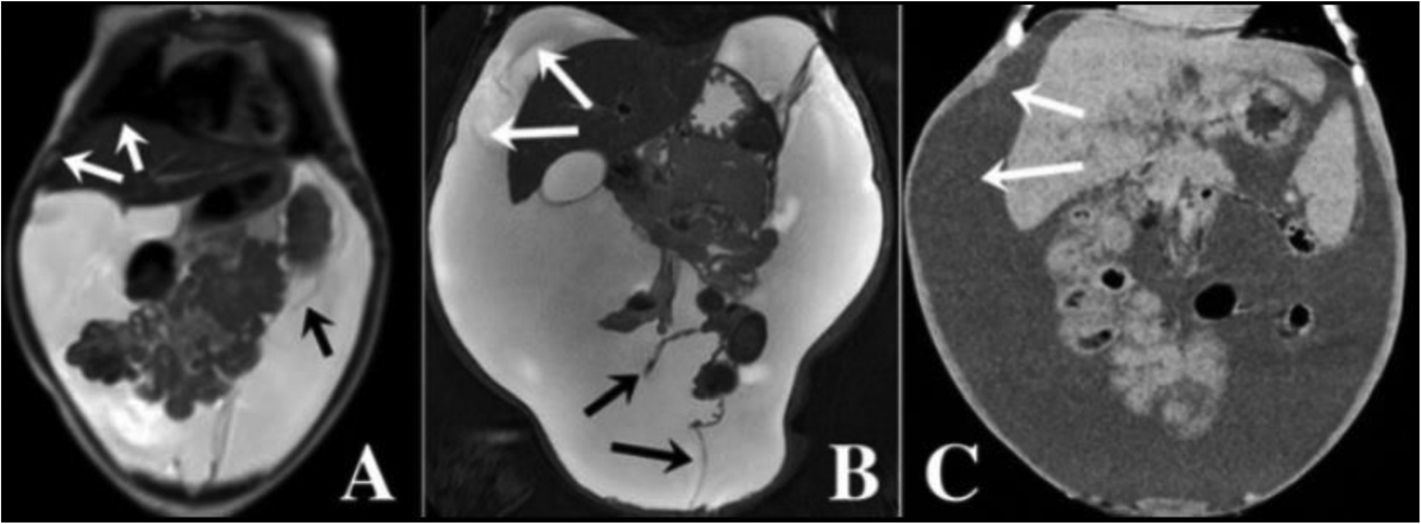
Radiological signs evoking pseudo-ascites. **a** Initial MRI of the patient: Coronal aspect showing distribution of the abdominal fluid and the sparing of the hepatodiaphragmatic space. Note the centralization of the bowel and the confusing floating aspect of the bowel loops, and the partial septae. **b** MRI performed at a later stage in the patient: Coronal aspect showing an increased amount of fluid that distributes in the whole cavity and do not spare the hepato-diaphragmatic space. **c** as a comparison, CT coronal aspect of free massive ascites in a child with decompensated biliary cirrhosis. (White arrows pointing at the hepato-diaphragmatic space – Black arrows pointing at partial septae)

**Fig. 2:**
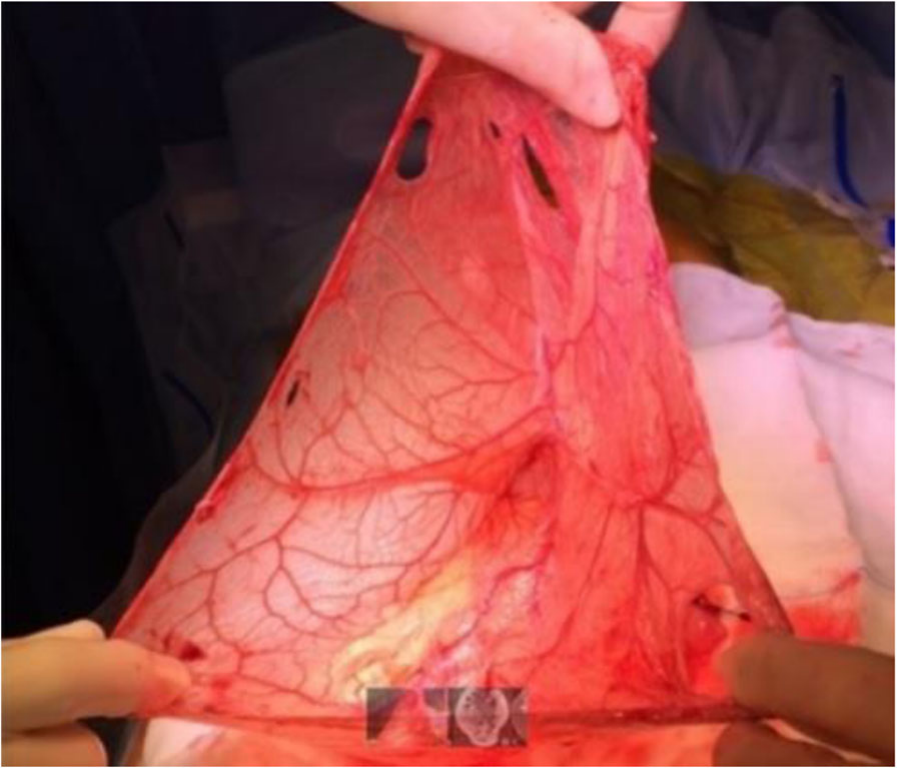
Intraoperative aspects. Intraoperative aspect of the cyst after its emptying, laparoscopic mobilization and exteriorization through the umbilical access site

A 7-month-old male infant was referred to our tertiary-care hospital for evaluation of a one-month history of progressive painless abdominal distention. His prenatal and familial history was negative, and his personal medical history included a surgical correction of bilateral inguinal hernia and thalassemia minor diagnosis. He appeared well, and his growth and neurological development were appropriate for chronological age. Physical examination was normal except for a distended and tense abdomen, painless on deep palpation, with a positive fluid-thrill; there was no evidence of organomegaly or abdominal masses. There were no clinical, laboratory or imaging signs of portal hypertension or liver disease. Abdominal ultrasound examination (US) showed the presence of fluid in the Morison’s pouch, in the left upper abdominal quadrant, and Douglas space; the liquid appeared free in the abdomen in the absence of definite septation nor a multi-cystic appearance thus confirming the clinical impression of ascites. There were no abdominal masses or abdominal organ abnormalities. A comprehensive workup excluded hepatic, renal, cardiac, intestinal, pancreatic, inflammatory or infectious diseases, malignancy, and congenital metabolic diseases. The child had had in the past a mild pleuro-pericardic effusion during a febrile illness and a transient mild hyperkaliemia, evoking a possible stomatocytosis due to *PIEZO1* mutation; the analysis of *PIEZO1* gene was however found negative. As an interferon-gamma test (QuantiFERON®) was positive, peritoneal tuberculosis was suspected, but the chest x-ray and the Mantoux test of the patient and his parents were negative. Gram and acid-fast stain and culture of three gastric aspirates resulted negative. Furthermore, the repeated interferon gamma test was negative and atypical mycobacterial infections were also excluded. A MRI of the abdomen only confirmed an abundant collection of liquid in the abdomen, with centralization of the bowel loops and a normal aspect of all abdominal organs (Fig. 1 a). Biochemical analysis of ascites revealed pale-yellow serous fluid with a normal value for the serum albumin-ascites gradient (gradient = 1.1). Triglycerides and total leukocytes counts were in the normal range (98% lymphocytes). At cytological examination, abdominal fluid appeared as a clear yellowish liquid that contained reactive lympho-monocytes, mesothelial cells, isolated foam cells and absence of malignant cells. Notably, the abdominal fluid rapidly re-accumulated just a few days after this first paracentesis. To promote a negative sodium balance and possibly reduce ascites production, symptomatic palliative diuretic management was started, by combining spironolactone (3 mg/Kg/daily) and furosemide (1 mg/Kg/daily). The child was then stable for a short time, but abdominal distension returned causing discomfort and breathing difficulty: multiple paracenteses were necessary during the following months. As repeated imaging and fluid analysis did not bring any new elements to orientate towards a precise diagnosis or treatment, a malformative pattern was also evoked and ruled out. Eighteen months after the onset of symptoms, in the absence of a positive diagnosis and of any improvement of the condition, and given the normality of all diagnostic tests, the initial diagnosis of ascites was reconsidered and pseudo-ascites was hypothesized. US and MRI studies were repeated but could not bring new elements, nor support the diagnosis of a PA (Fig. 1 b). At that point, because the ascites was refractory to treatment with recurrent need for hospitalizations for paracentesis and associated with a poor quality of life, surgery was proposed. The plan was to proceed with a diagnostic laparoscopy to confirm the free ascites and the absence of any other precise condition, followed by the creation of a peritoneo-venous shunt [3] if free and cryptogenic ascites was confirmed. Laparoscopy led to the identification of a benign giant cystic lymphangioma of the omentum, which was mobilized laparoscopically and radically resected through the umbilical site of the laparoscopy access. The cyst covered all abdominal organs but none was intimately involved and the resection was extended to an en-bloc resection of the great omentum (Fig. 2). There was no free fluid in the abdomen. The resolution of symptoms was immediate, and there has been no recurrence of abdominal distention: the child is now doing fine one year after surgery. The anatomo-pathological study confirmed the surgical diagnosis.

## Discussion

PA is defined as an abdominal distention mimicking ascites. When PA is caused by large abdominal cysts, it is difficult to distinguish from simple ascites because of the presence of an ultra-thin-wall and large liquid collection. It is a very rare entity and thus the literature on this argument is scarce: it mostly comprises case reports and small case series. Vrettos and colleagues recently reviewed this topic emphasizing the challenge and the difficulty in positively identifying a PA [1]. Their review shows that most of the times, the correct diagnosis is reached months - or years - after the first clinical presentation; often the main limit for a timely diagnosis is having not considered the PA in the differential diagnosis. Omental and mesenteric cysts are very rare intra-abdominal masses with an estimated incidence of 1/140.000 children and only few reports are found in literature. Cystic lymphangioma is one of these benign cysts that may grow large enough to mimic ascites [2, 4]. It usually presents in male children under the age of 12 years. Macroscopically, cystic lymphangiomas may be simple and gigantic, or may consist of multiple cysts of various size. Their lining is composed of flat endothelial cells with a wall containing smooth muscle fibers, lymphoid tissue, and occasionally foam cells. Because these walls are extremely thin, they may not be identified at US or other imaging, as in our case. Intraperitoneal cystic lymphangiomas are partly free in the peritoneal space, but may also adhere to other abdominal structures, which may render their radical resection more difficult and expose to the risk of recurrence. Differentiating a huge lymphatic cyst from ascites is challenging, as its walls are so thin that they are not identified on imaging in many cases, giving the false impression of a free ascites. The typical radiological signs of a PA – i.e. the fact that the loops appear conglomerated in the center of the abdomen, or the fact that there may be aspects of septations in the fluid collection – are not a constant finding at imaging and also they are not specific [2, 4–6]. In this case, the imaging was confusing because bowel loops were centralized but not conglomerated as one expects in free ascites (Fig. 1b). In addition, although some septations were identified on MRI and US, they occupied partially the abdomen because the cyst was single and huge: it was not possible for imaging to conclude for the presence of a cystic and loculated mass (Fig. 1a, b). Another radiological sign is the way the fluid is distributed while it accumulates in the peritoneal cavity. A free ascites occupies any disposable space (firstly the Morison’s pouch and the Douglas space, then the upper abdominal quadrants and the whole cavity) separating progressively any organ from the abdominal wall. There are however some specific areas - i.e. the space between the diaphragm and the liver – where a free ascites is found and which distinguishes the latter from a large abdominal cyst - that usually does not extend in that space (Fig. 1a). This sign is however not absolute as gigantic cysts may also separate the liver from the diaphragm [6]. Interestingly, in our case, although a first MRI showed the absence of liquid between the liver and the diaphragm, this sign disappeared with time when the abdominal distension worsened, and the liquid distribution mimicked exactly that of free ascites at the subsequent MRI (Fig. 1b). Overall, even with most modern imaging techniques, a PA can be mistaken for ascites. The most important clue for a timely diagnosis is a high index of suspicion, and considering the diagnosis of PA even if the imaging is not typical. A PA should be considered in particular when all other conditions have been excluded. This case report is a call for an increased awareness of pediatricians, gastroenterologists and other clinicians who are dealing with patients with apparently refractory ascites.

## Conclusions

Although PA is often amenable to a simple and radical treatment, it remains a challenging condition to identify and it is typically associated with unnecessary delays before a diagnosis and cure are proposed. This retrospective case description should increase the awareness of clinicians for that condition. It also suggests that the absence of liquid between the liver and the diaphragm is a specific, although not pathognomonic, element at imaging that may help to differentiate PA from free ascites.

## Data Availability

Not applicable.

## Abbreviations

CT: computed tomography
MRI: magnetic resonance imaging
PA: pseudo-ascites
US: ultrasound
